# Managing Everyday Life: A Qualitative Study of Patients’ Experiences of a Web-Based Ulcer Record for Home-Based Treatment

**DOI:** 10.3390/healthcare2040492

**Published:** 2014-12-15

**Authors:** Marianne V. Trondsen

**Affiliations:** Norwegian Centre for Integrated Care and Telemedicine (NST), University Hospital of North Norway (UNN), P.O. Box 35, N-9038 Tromsø, Norway; E-Mail: marianne.trondsen@telemed.no; Tel.: +47-4151-0792; Fax: +47-7775-4099

**Keywords:** ulcer care, e-health, teledermatology, web-based record, home-based treatment, professional-patient interaction, empowerment, qualitative study

## Abstract

Chronic skin ulcers are a significant challenge for patients and health service resources, and ulcer treatment often requires the competence of a specialist. Although e-health interventions are increasingly valued for ulcer care by giving access to specialists at a distance, there is limited research on patients’ use of e-health services for home-based ulcer treatment. This article reports an exploratory qualitative study of the first Norwegian web-based counselling service for home-based ulcer treatment, established in 2011 by the University Hospital of North Norway (UNN). Community nurses, general practitioners (GPs) and patients are offered access to a web-based record system to optimize ulcer care. The web-based ulcer record enables the exchange and storage of digital photos and clinical information, by the use of which, an ulcer team at UNN, consisting of specialized nurses and dermatologists, is accessible within 24 h. This article explores patients’ experiences of using the web-based record for their home-based ulcer treatment without assistance from community nurses. Semi-structured interviews were conducted with a total of four patients who had used the record. The main outcomes identified were: autonomy and flexibility; safety and trust; involvement and control; and motivation and hope. These aspects improved the patients’ everyday life during long-term ulcer care and can be understood as stimulating patient empowerment.

## 1. Introduction

Chronic and complex ulcers are a widespread problem that represents a significant challenge for patients and health services [1,2]. Chronic ulcers are painful and impair mobility and activity, which reduces patients’ quality of life [3,4]. Community nurses, in collaboration with general practitioners (GPs), have the primary responsibility for providing care to patients with ulcers, but patients’ self-care can play an important role. However, insufficient knowledge of ulcer treatment is common in primary healthcare services, as well as in local hospitals [1,2,5]. Treatment of long-lasting ulcers often requires the competence of a specialist, access to which is limited in rural areas. Repeated visits to specialist hospitals are often necessary, representing an important cost factor for healthcare systems and placing a significant burden on the patients, because such visits are time-consuming and often include long travel distances [6,7].

Teledermatology, which is defined as delivery of dermatology services through the use of information and communication technology (ICT) [8], is a rapidly evolving field that offers great potential for long-term ulcer care and contributes to overcoming inequities in access to health services across geographical regions [5,6,7,8]. Videoconferences, cameras and mobile devices, as well as web-based electronic transmission of digital photos and written information make it possible for ulcer experts from specialist hospitals to offer opinions for diagnosis and recommendations for treatment without delay [6,9]. The expert advice can be delivered in real time or in store-and-forward systems to GPs, community nurses, physicians or nurses at local hospitals, as well as directly to patients in-home.

Teledermatology has provided high levels of concordance in diagnosis and management when compared with face-to-face consultations [10]. It is cost-effective, providing rapid evaluation and treatment, as well as more efficient use of human resources [5,7]. Access to ulcer experts through web-based tools also enhances community nurses’ knowledge of ulcer treatment [5,11]. A study by Binder* et al.* (2007) demonstrated that web-based follow-up of ulcer care, which included sharing clinical information and digital photos between community nurses and ulcer specialists, improved the quality of treatment [6]. A good healing rate was achieved, and patients, community nurses and ulcer specialists were all satisfied with the service [6]. Nevertheless, the development of teledermatology as a routine service remains limited [8].

This article reports an exploratory qualitative study of the first Norwegian web-based counselling service for home-based ulcer treatment (www.pleie.net). In 2011, the Department of Dermatology (DoD) at the University Hospital of North Norway (UNN) established a web-based ulcer record system in collaboration with the Norwegian Centre for Integrated Care and Telemedicine, using an adapted web-based ulcer record system developed by Danish Telemedicine [9,11]. The purpose of the web-based counselling service was to optimize treatment and care of complex and demanding ulcers through self-management of home-based ulcer treatment. Use of the web-based service in home-based treatment was to be a part of the follow-up of ulcer patients at UNN, in combination with clinical consultations at the DoD. The web-based ulcer record (www.pleie.net) is a store-and-forward system that consists of databases, an application to communicate digital photos and text between the users and a tool to measure and analyze ulcers. The log-on procedure for the record system requires a two-factor authentication, employing a username, password and a one-time password sent by SMS from the server to the phone number registered in the user profile, and gives access only to the ulcer records of the patients that the user is authorized to access [9]. Patients or community nurses take photos of the ulcers with a digital camera or a mobile phone and publish the digital photos, leave comments or address questions in the web-based record to a specialist ulcer team consisting of specialized nurses and a dermatologist at the DoD. The nurses on the team have the primary responsibility of replying as quickly as possible and within 24 h. The communication between the user’s web client on a mobile phone or a computer and the ulcer record system is encrypted. The system runs in a secure environment. All of the photos, comments and responses are archived in the record for retrospective examination. The measurement tool enables an analysis of the ulcers’ development and presents diagrams for observing changes over time. This article addresses patients’ experiences of using the web-based ulcer record for their home-based ulcer treatment without regular assistance from community nurses or other community health services.

Most of the studies of teledermatology have been on the effects and outcomes from a medical perspective. Thus, there is a need to supplement this research by exploring patients’ experiences. Web-based applications make it possible for patients to get direct access to expert advice, but little is known about patients’ attitudes towards using a web-based record to support their home-based ulcer treatment. Patients’ perspectives have, to a large extent, been left out of studies on telemedicine and e-health applications, even though such services often constitute a new practice in which patients are expected to play an active role [12,13,14].

Recently, there has been a shift in Western healthcare towards embracing patient-centered care, as well as prioritizing the development of patient-oriented ICT [15,16]. The empowered patient, characterized as active, engaged and informed in health matters, is a ubiquitous ideal in contemporary healthcare [15,17]. Self-management interventions are highly valued [18]. Patient empowerment is argued to result in positive health outcomes; in particular, there are optimistic expectations of the empowerment potential in patients’ use of e-health [15,19]. As pointed out by Andreassen (2012), however, in e-health policy and research, the question of the empowerment potential of e-health has been limited to a focus on information gathering through ICT [15]. This is expected to increase patients’ involvement and control, as well as to stimulate democratization of power relations in the patient-health professional relationship [15]. However, patients’ use of technology in healthcare is multifaceted and might also load patients with new tasks, responsibilities and obligations [12]. Hence, limitations and uncritical use of the notion of empowerment have recently been discussed [20,21]. More empirical research is needed that enables a nuanced approach to the complexity of e-health communication between patients and professionals and how the technology is integrated into patients’ everyday practices [14,17,22,23]. Accordingly, this study explores patients’ experiences of using a web-based ulcer record on their own for home-based treatment, and the empirical findings will be discussed in light of the empowerment debate.

## 2. Methods

The study was exploratory in nature, and a qualitative research design was used. Based on semi-structured interviews with ulcer patients, community nurses and the ulcer team at the specialist hospital, the study explores experiences of using a web-based record (www.pleie.net) as a tool for home-based ulcer treatment from different angles. This article, however, is based on interviews with the ulcer patients who had used the web-based record in their home-based ulcer treatment without regular assistance from community nurses. The health professionals’ experiences with using the web-based record in ulcer treatment, the processes entailing use and the enacted improvements of care are reported in previous [24] and forthcoming papers [25].

### 2.1. Participants

Four patients voluntarily participated in the study and were recruited through the DoD. Although the small number of patients limits the study, these four participants represented the total population of patients who had been offered access to the web-based ulcer record based on selection by the specialist ulcer team at the DoD during the study period. The ulcer team offered access and follow-up through the web-based record for two groups of users; patients who were capable of taking care of their home-based ulcer treatment assisted by the record (without regular assistance from community nurses) and community nurses doing the ulcer treatment for patients who were not capable of managing the ulcer care by themselves. The researchers were not involved in the selection of patients who were offered the web-based ulcer record. Hence, the four patients who were offered access and who used the web-based ulcer record were all included in the study and interviewed. The researchers did not have access to patients who potentially could have refused the use of the record. Although the recruitment strategy is not investigated in this study, it is worth noting that the study was initiated at an early stage of the implementation process of the new e-health service. This may be one of the reasons why there were few patients recruited. Thus, we found it important to address these patients’ experiences of use of the web-based ulcer record in order to increase knowledge for the development of this new web-based counselling service. The study participants were 40 to 51 years of age, two men and two women. All of them lived a long distance from the specialist hospital; three lived 750 to 850 km away. The duration of ulceration ranged from five months to three years. They all had complicated ulcers and were still receiving treatment from the DoD at the time of the interviews. Due to other health problems, one of the patients was a disabled pensioner and another was working half-time. The other two were not capable of working because of their ulcers. All of the patients had access to a computer and the Internet at home. They were given both written information and practical training from the DoD in how to use the web-based ulcer record before they started to use the counselling service at home.

### 2.2. Data Collection and Analysis

The interviews were conducted from September to December, 2011. Three of them were carried out in a meeting room at the hospital. The fourth interview was performed at the interviewer’s office. The author did three of the interviews, and the project manager of the study did the fourth. A semi-structured approach was taken. The interviewees were first asked a broad open question, to “tell their story” in any way they chose [26]. Then, an interview guide consisting of a list of questions related to the web-based record and their experiences as ulcer patients was consulted. Probing questions were added to gather further details [27]. The interview guide was shaped in collaboration between the two interviewers. The interviews lasted between 45 and 75 min. Three out of four interviews were digitally recorded and transcribed, while notes made by the interviewer were used to document the fourth interview. The notes were approved by the interviewee during and after the interview.

The digital recordings of the interviews were transcribed verbatim and were analyzed by the author following an inductive, issue-focused approach [28], focusing on emerging themes, events or processes introduced by the participants. The aim was to explore issues raised in the interviews rather than differences between individuals. Throughout the issue-focused analysis, the data material was coded, sorted and integrated [28].

### 2.3. Ethics

The Committee for Medical Research Ethics of Northern Norway approved the study. Permission to conduct the interviews was obtained from the manager of the hospital dermatology ward (DoD), and the ulcer team gave information about the study to potential participants. The patients, who gave their voluntary consent to participate, were informed that they could withdraw from the study at any time. To ensure patient confidentiality, pertinent biographical details were concealed in the quoted material.

### 2.4. Validity and Reliability

Two researchers collaborated in carrying out the study, and the author performed the analysis of the interviews with the patients. However, to assure trustworthiness, the project manager read the transcripts and reviewed the author’s codes, categories and interpretations during the analysis process [29]. Work-in-progress drafts of the paper were presented in a workshop and discussed with the project group and research colleagues. Verbatim citations from the participants were included to ensure reliability by low-inference descriptors [30].

## 3. Results

From the analysis of the interviews, four recurring interrelated aspects were identified as crucial with respect to the patients’ use of the web-based record in their home-based ulcer treatment: (1) autonomy and flexibility; (2) safety and trust; (3) involvement and control; and (4) motivation and hope.

### 3.1. Autonomy and Flexibility

All of the patients emphasized the saving of time and the avoidance of the need to travel to the DoD as the main advantages of the web-based record. All of them had to travel about half a day by boat or plane (including plane changes or intermediate landings) to visit the hospital for control or treatment. These journeys could be required two or three times a month. Often, they had to arrive the day before their appointments, and sometimes, they were hospitalized for several days. One patient needed an assistant with him in order to make the journey. With fewer visits to the specialist hospital, the patients experienced increased autonomy in daily life.

One of the patients expressed enthusiastically:

It is great! I wouldn’t be without the ulcer record because it’s so easy. It’s incredible—I can sit at home and do it! I don’t have to travel and spend time on that. … Well, I think it’s simply fantastic!

Another patient stated that fewer journeys made it possible to live a more normal life:

I don’t need to travel to the hospital and have been able to live a normal life all the time. When problems occur, I just log on. You don’t have to leave the house. It’s very positive.

Three patients had previously received help several days a week from outpatient clinics at a local hospital or primary healthcare center, for treatment or to change bandages. Guidance from the ulcer team at the DoD through the web-based record made it much easier for the patients to take care of their ulcers at home on their own. In all, the patients emphasized improved quality of life because they had more time for family, work, leisure activities and social life.

In addition, the patients emphasized that the web-based record provided more flexibility in managing ulcer treatment. They were allowed to choose when, where and how often communication with the ulcer team would take place. With a web-based record available 24 h a day, seven days a week, they could post photos and messages and read the answers when it was convenient. One patient commented:

It is easier for me; I am not put under stress. I can enter the record whenever I feel like it. If something occurs, I can write whenever I want.

### 3.2. Involvement and Control

Another recurring theme expressed by patients was that use of the ulcer record made them feel more involved in assessments and treatment. Communication between the patients and the ulcer team was primarily initiated by the patients. They decided how, when and how often they wanted to send photos. Along with the photos, they often wrote a comment about what they assumed was adequate for further treatment. They also had to be alert for aggravation of the ulcers and to determine when they needed help from specialists. One of the participants highlighted patient involvement as an important aspect of the web-based record:

The ulcer record is very good because you are personally involved. It’s really a good thing. You have to concentrate; you have to follow up by yourself and have an interest in it to succeed.

Some patients reported that using the web-based record provided a feeling of being in control. It facilitated access to complete information about their illness for the first time in their relatively long illness career:

I feel that I have much more control because of the tight follow-up through the ulcer record. … It is perhaps one thing that health personnel often forget; they don’t realize that there is little information coming down to you as a patient. Now, I have that information, now I understand what they are thinking, and now I get feedback. It is very important.

All information about their ulcers was documented, archived and available in the record for the patients. As one patient stated, “The information is not going over my head anymore”.

Others said they sometimes used the ulcer record as an archive for reviewing what the ulcer team had said and what kind of treatment was done:

I think it’s very good to have everything written in the record because then I can go back later and see what she (the ulcer nurse) actually said about that, instead of sitting in a dialogue, and then just thinking afterwards, “What is it we actually agreed on?” So, I think it’s very good.

Overall, patients felt they gained more ownership and control of their illness, as well as more knowledge on ulcer treatment. Being knowledgeable gave them the ability to manage their ulcers and contributed to a sense of self-confidence in their at-home treatment.

### 3.3. Safety and Trust

All of the patients had previously been in contact with a range of primary healthcare services and health professionals, but had experienced a lack of competence and continuity in treatment. Hence, the patients felt safer when being followed up by ulcer experts at the DoD. They stressed that the 24/7 access to the ulcer team through the web-based record provided an increased sense of safety and trust:

I feel confident, and I trust the system. Because … I send photos and tell them what I have put on the ulcer and they read it and see how the ulcer looks. I describe it and get feedback immediately, with advice like “Now you can do this, not do that, or do this instead of something else”. This response has been great. And, I also know that they answer very promptly!

This open gateway to expertise was mentioned as a source of security, because patients could rapidly contact an ulcer nurse if their ulcers worsened:

I’m really privileged! Instead of having to wait for at least 14 days to receive help, I now get it immediately. That is fantastic, and it is reassuring me and makes me feel secure.

Another consideration that supported their sense of safety and trust was that the ulcer team made their medical assessments based on photos and written texts about the ulcers, not only on oral descriptions by telephone:

It’s clear; they [the ulcer nurses] have something to look at, right. And, I have received such good instructions from them.… I am much safer than before—confident that my foot won’t “fall off” at worst. … I receive feedback; in fact, it is professionals who are looking at the ulcers. This makes me feel safe.

For their own sense of security, some patients chose to send photos after every bandage change. The patients said they felt calmer after sending photos and comments to the ulcer team, and they used the web-based record to share or transfer responsibility for their treatment to the professionals.

In addition, communication through the web-based record was with the same persons who followed up on their ulcers face-to-face at the hospital, which inspired trust. Patients appreciated that only a few professionals, who had complete information about their illness history, were involved in their ulcer treatment. One of the patients stressed that the “person on the other end of the record” who looked at the photos and gave feedback was an ulcer nurse she trusted and knew very well. She appreciated that she did not need to continuously tell her “illness story” to new nurses and doctors.

### 3.4. Motivation and Hope

Finally, the patients emphasized that the follow-up from the DoD through the web-based record had sparked new motivation and a more optimistic attitude towards becoming healthy again. The patients had been suffering from their complex ulcers for several years and said they had found it hard to be optimistic about the healing process. They described earlier periods when they were discouraged and afraid of the potentially serious consequences if their ulcers did not heal. Using the record provided a sense of doing something besides just waiting. The immediate responses from the ulcer team made it possible to make rapid modifications to treatment procedures if needed. By looking back at the archive of photos and using the measurement function, patients were able to visualize the status of their ulcers. This visualization provided an opportunity to compare the ulcers historically and to see the progress that otherwise would have been impossible to see in daily treatment:

I used to go back and look at the photos to see how bad it has been. There was a period when I felt nothing was happening and there was no progress at all. … But, when I logged on, went back and had a look at the photos, I saw that something had happened after all. So, it was very good. I have also tried the chart to see if and how the ulcer has been reduced. It was funny because I saw that the ulcer had surprisingly been reduced!

## 4. Discussion

The patients only described advantages with the use of the web-based record, which were linked to four interrelated aspects: (1) autonomy and flexibility; (2) involvement and control; (3) safety and trust; and (4) motivation and hope. These outcomes of their use of the web-based ulcer record represented changes that improved the everyday lives of patients with long-lasting ulcers.

First, the patients emphasized that the practical and timesaving aspects of using the web-based record for ulcer treatment contributed to more autonomy and flexibility in their lives. In line with previous studies of teledermatology [6,7,10], the patients found that the web-based follow-up of the ulcers reduced repeated visits to the hospital dermatology ward. Moreover, they appreciated staying at home, where they could adjust and integrate the ulcer treatment into other daily activities. The patients could have contact with the ulcer team through the record whenever it suited them or whenever they considered it necessary. Accordingly, Dedding* et al.* (2011) have pointed out that not having to wait for an appointment or to organize dialogues that are otherwise difficult to arrange within traditional healthcare are features of e-health that can be understood as supplementary to existing forms of care [23]. In the study presented here, patients experienced the web-based record as increasing their autonomy with respect to managing their illness.

Moreover, the patients emphasized how their use of the web-based record provided more involvement and control in the ulcer treatment, as well as increasing their knowledge of ulcer care, not only through the flow of actual information and recommendations, but also by the act of using the record for themselves. The archived information and photos made it possible for patients to read their illness history and recall what had been previously done and recommended. The patients compared photos of their ulcers over time, used the measurement instruments in the record to analyze their ulcers and made continuous assessments of the healing process. Interaction through the ulcer record was also primarily initiated by the patients. They often suggested new procedures for treatment based on their own experience. Accordingly, the patients’ use of the web-based record can be described as participatory and empowering, stimulating them to be informed and involved. Dedding* et al.* (2011) have shown how patients’ use of e-health can foster patient empowerment by providing interaction with health professionals, offering information that supports choices and presenting a decentralized decision-making structure [23]. Increased knowledge and extended control over health matters are basic elements of an empowering process [20].

However, the web-based record facilitated a constant gateway to ulcer specialists. The patients reported that the use of the record increased a sense of safety and trust in the healing process. Access 24/7 to expertise and the fact that the ulcer team’s assessments were based on visual documentation acted as important sources of security for the patients. This functionality of the web-based record was considered particularly important because of patients’ earlier experiences of long-lasting and challenging healing processes and fears of actual consequences if their ulcers would not heal. A common fear expressed by patients with long-lasting leg ulcers is the risk of amputation [3]. The reassuring aspect of new e-health tools has also been found in other studies: an e-health service for eczema represented a “safety alarm device” for the users by facilitating rapid contact with medical professionals if needed [14].

The patients gained a sense of relief by frequently sharing photos of their ulcers with the ulcer team. It made them feel that the responsibility for their treatment was shared or transferred to ulcer specialists. This is much in line with Henwood* et al.* (2003), who illuminated the constraints to the emergence of the “informed patient or empowered patient”, in situations where patients also want health professionals to take responsibility and be in charge of their healthcare [17]. Although patients want to be involved and informed in all facets of their illness, transferring parts of the responsibility for treating their illness to their doctor is an important aspect of medical consultations [14]. As indicated by others [12,23], introducing new technology to the patient-healthcare provider relationship places new responsibilities on the patient and encourages patients to act for themselves in their healthcare. The patients in our study had to continuously inspect and assess their ulcers and decide when it was necessary to send photos or consult the ulcer team through the record. A previous study of electronic communication between patients and GPs demonstrated that patients tend to use technology in a way that transfers responsibility back to their doctor [31].

However, the patients here did not give away the responsibility for their treatment to experts. Their communication with the ulcer team through exchanges of digital photos, comments and responses in the web-based record can instead be understood as collaborative. The patients’ experiential knowledge of their ulcers and the expert knowledge from the ulcer team together constituted the basis for further treatment. This patient-professional collaboration is consistent with what has been noted elsewhere: that patients’ use of e-health services might induce a shift away from professionally-led interaction to a patient-provider partnership [23].

Finally, the patients were enthusiastic about the web-based record as a tool that stimulated their motivation to “keep up their spirits” in the healing process and to maintain hope for the future. Through visualization using digital photos and measurement applications, the patients could observe the healing process of their ulcers, which they could not see with their naked eyes during day-to-day ulcer care. Patients’ motivation and involvement in their ulcer treatment are crucial factors in the healing process in cases where patients are caring for their ulcers at home without assistance from community nurses.

These different outcomes of the ways patients used the web-based record shed light on the diversity and complexity of patients’ experiences of e-health services in everyday life. The patients collaborated with the ulcer team by being informed and involved. However, the record is more than a gateway to medical information and expert assessments. It also adds autonomy and flexibility in everyday life and increases patients’ sense of safety, trust, motivation and hope. At the same time, the patients were both taking responsibility for their own health and transferring responsibility back to the specialists. The diversity of patients’ experiences raises questions about whether the notion of empowerment can capture the complex and sometimes contradictory impacts of e-health services in patient settings. Access to e-health technology can have both empowering and disempowering elements, related, for example, to responsibility. Dedding* et al.* (2011), in a literature review of the consequences of e-health, found that the relationship between technology and the patient-provider relationship is more complex than the often polarized responses indicated [23]. E-health has diverse and potentially contradictory effects on the patient-professional relationship in healthcare [23]. In addition to its informative potential, patient-oriented ICT can affect other important aspects of patients’ experience, such as safety and trust [15].

The impacts of long-lasting ulcers on the quality of life are well documented [3,4], and patients in our study emphasized a range of limitations and disruptions to their mobility, activity, self-esteem and social life. The concept of “biographical disruption” [32] illustrates such disruptive aspects in life when a chronic or long-lasting illness occurs. Thus, the patients experienced that use of the web-based record strengthened autonomy, flexibility and independence in their everyday lives. We can interpret the web-based record as providing these patients with a tool for reorientation of earlier disruptions in daily life in the direction of normality. When they became ill with long-lasting ulcers, they had to adjust their lives to the illness. With access to the web-based record, they could, to a greater extent, adjust the illness to their everyday lives, perhaps giving less attention to their ulcers and their role as patients. The web-based record also stimulated a revitalization of their lifestyle with long-lasting ulcers, which might have provided motivation and hope for a brighter future. Transforming the spiral of hopelessness and learning to manage the situation are major components in furthering the healing process [3]. Thus, as proposed by others [12,14,23], there is a need to examine patients’ experiences and everyday practices when new e-health services are introduced in order to illuminate the nuances and diversity in e-health communication in patients’ efforts to manage health issues.

Although this is an exploratory study with the total population of patients included, a limitation of the study is the small number of participants. However, we have explored a new e-health tool for ulcer treatment, and it is a novel study addressing patients’ experiences of using a web-based record in home-based treatment without support from community nurses. The qualitative design facilitated in-depth insight into these four patients’ experiences and some of the roles a web-based record could play in the everyday lives of patients with long-lasting ulcers. Thus, the findings could provide a useful starting point for broader analyses of interactions between patients and health professionals through web-based records for ulcer treatment.

## 5. Conclusions

Through the introduction of a web-based ulcer record for home-based ulcer treatment, patients gained improved access to expert advice from a hospital dermatology ward as part of a collaborative follow-up for ulcer treatment. Although this e-health tool placed new responsibilities on patients, they used it in ways that can be interpreted as stimulating patient empowerment. Introduction of the web-based record not only provided patients with access to medical information and expert recommendations, but also made it possible to better integrate ulcer treatment into their daily lives and increased patient autonomy and flexibility. This might be crucial in strengthening patients’ own efforts, involvement and motivation to “keep up their spirits” while managing the healing process and on-going ulcer treatment. Hence, the web-based record can be understood as facilitating efforts for a reconstruction of normality in patients’ everyday lives after the disruption caused by their ulcers.

Focusing on patients’ perspectives, this study has shed light on their use of a web-based ulcer record and on multiple advantages of this technology for their everyday lives, which were important for managing long-lasting ulcers. We can question whether the concept of empowerment provides a complete picture of the complexity of e-health technologies as used by the patients. It might be necessary to extend, or go beyond, the empowerment concept, to capture the diversity and abundance of impacts of this technology, mainly supporting patients’ management of everyday life. Discussing additional or contradictory experiences of patients is an important issue for further research addressing how e-health services may support proper treatment for ulcer patients.
